# Efficacy and safety of tofacitinib in ankylosing spondylitis patients: a systematic review and meta-analysis

**DOI:** 10.1016/j.clinsp.2026.101147

**Published:** 2026-09-09

**Authors:** Le Shen, Kang Han, Lei Bo, Fei Huang

**Affiliations:** a Department of Orthopedics, The 960th Hospital of the PLA Joint Logistics Support Force, Shandong, China; b Department of Clinical Laboratory, The 960th Hospital of the PLA Joint Logistics Support Force, Shandong, China

**Keywords:** AS, Tofacitinib, Meta-analysis, RCT, Cohort study

## Abstract

**Background:**

Tofacitinib, an oral Janus Kinase (JAK) inhibitor, exhibits promising therapeutic potential for Ankylosing Spondylitis (AS). This meta-analysis aimed to assess its efficacy and safety in AS patients.

**Methods:**

PubMed, Embase, Web of Science and Cochrane Library, were systematically searched for relevant studies until May 21, 2025. Literature screening was conducted using EndNote 21. Risk of bias for encompassed Randomized Controlled Trials (RCTs) was examined via the Cochrane Risk of Bias tool (RoB2.0). The quality of cohort studies was rated using the Newcastle-Ottawa Scale (NOS). Meta-analysis was performed via Stata 18.0.

**Results:**

Five studies were encompassed: three RCTs and two cohort studies. Regarding primary efficacy outcomes, tofacitinib significantly increased response rates for ASAS20 (Relative Risk [RR = 1.96]; 95% Confidence Intervals [95% CI: 1.74‒2.21]), ASAS40 (RR = 1.88; 95% CI: 1.48‒2.40) and ASAS-PR (RR = 2.55; 95% CI: 1.82‒3.58). Regarding Disease Activity (DA), tofacitinib demonstrated significant benefits in achieving BASDAI50 (RR = 1.90; 95% CI: 1.64‒2.20) and ASDAS. Physical Function (PF) and Quality of Life (QoL) outcomes were also notably ameliorated in the intervention group. Subgroup analysis indicated that 5 mg dosage and 16-week treatment duration exhibited the most favorable efficacy trends across most outcomes. Overall rates of Adverse Events (AEs) and Serious Adverse Events (SAEs) were similar between tofacitinib and placebo groups. However, the risk of Upper Respiratory Tract Infection (URTI) was significantly higher in the tofacitinib group (RR = 2.15; 95% CI: 1.27‒3.63).

**Conclusion:**

Tofacitinib is an effective and generally well-tolerated active AS therapy, significantly ameliorating DA, PF, and QoL, although it may elevate the risk of URTI.

## Highlights

• Tofacitinib improved ASAS response in ankylosing spondylitis patients.
• It ameliorated disease activity, physical function, and quality of life.
• 5 mg dose and 16-week treatment showed most favorable efficacy trends.
• Safety similar to placebo except for higher risk of upper respiratory infection.

## Introduction

Ankylosing Spondylitis (AS), or radiographic axial spondyloarthritis,^1^ a persistent inflammatory rheumatic disease, mainly affects the axial skeleton and causes joint stiffness and spinal deformities.^2^ Its clinical manifestations also encompass peripheral arthritis, enthesitis, and extra-articular features like uveitis, psoriasis, and inflammatory bowel disease.^3^^,^^4^ The prevalence of AS varies geographically, ranging from 6.5 to 540 per 100,000 individuals.^5^ Non-Steroidal Anti-Inflammatory Drugs (NSAIDs) are advised as first-line therapy, followed by biologic Disease-Modifying Anti-Rheumatic Drugs (bDMARDs) for those displaying high Disease Activity (DA) in spite of the use of, or intolerance/contraindication to, two or more NSAIDs.^6^ Given that bDMARDs are administered intravenously and approximately 20%‒40% of the AS population exhibits intolerance or inadequate response to bDMARDs,^7^ there remains an unmet need for alternative oral therapies that are convenient to use and possibly operate via different mechanisms.

Tofacitinib is an oral Janus Kinase (JAK) inhibitor that has a key advantage over biologic agents, its oral route of administration, and has demonstrated substantial potential in treating AS. By selectively inhibiting the JAK1/JAK3 signaling pathways and blocking downstream signaling of multiple pro-inflammatory cytokines,^8^ tofacitinib modulates cytokines implicated in AS pathophysiology, like IL-17 and IL-23.^9^, ^10^, ^11^ Serum IL-17 level is typically elevated in the AS population,^12^ and antibodies targeting IL-17 are effective in treating AS.^13^ Tofacitinib also reduces serum TNF-α levels,^14^ thereby attenuating inflammatory responses. Furthermore, its efficacy and safety have been studied in autoimmune diseases like rheumatoid arthritis,^15^^,^^16^ suggesting its potential applicability in AS.

Phase II and III clinical trials have demonstrated that tofacitinib notably ameliorates symptoms in AS patients compared with placebo, with no new safety risks.^17^^,^^18^ The foregoing findings provide a novel oral AS treatment option. Nevertheless, systematic evaluation of its efficacy and safety is lacking. Our study endeavors to assess the effects of tofacitinib on core symptoms in the AS population, explore efficacy differences across various doses and treatment durations, and evaluate safety outcomes (e.g., infection risk) via a systematic review and meta-analysis, thereby providing evidence for new pharmacologic options in treating AS, particularly as an alternative for patients resistant to biologic therapies.

## Method

This review followed the Preferred Reporting Items for Systematic Reviews and Meta-Analyses (PRISMA) guidelines^19^ (Appendix 1) and was registered in PROSPERO before commencement in August 2025 (Registration n° CRD420251125225).

### Search strategy

MeSH and free-text terms were utilized to search PubMed, Embase, Web of Science, and the Cochrane Library before May 21, 2025. Moreover, the references of encompassed studies were manually checked. Major search terms encompassed: “AS”, “ankylating spondylitis”, “ankylopoietic spondylarthritis”, “ankylopoietic spondylitis”, “tofacitinib”, and “tasocitinib”. The strategies are detailed in Appendix 2.

### Eligibility criteria

Inclusion criteria were: 1) A randomized controlled trial (RCT) or cohort study; 2) Adult AS patients; 3) Studies that included a tofacitinib treatment arm and a concurrent control arm (e.g., a placebo group in RCTs, or the original placebo arm from the parent RCT in post-hoc cohort studies); and 4) Outcome measures of Assessment in Spondyloarthritis International Society (ASAS) 20, ASAS40, ASAS Partial Remission (PR), Bath Ankylosing Spondylitis Disease Activity Index (BASDAI) 50, Ankylosing Spondylitis Disease Activity Score (ASDAS) with C-Reactive Protein (CRP) Inactive Disease (ID), ASDAS Low Disease Activity (LDA), ASDAS Major Improvement (MR), ASDAS Clinically Important Improvement (CII), modified Bath Ankylosing Spondylitis Functional Index (ΔBASFI), modified Bath Ankylosing Spondylitis Metrology Index (ΔBASMI), modified Short Form-36 Health Survey Version 2 Physical Component Summary (ΔSF-36v2 PCS), modified Functional Assessment of Chronic Illness Therapy-Fatigue (ΔFACIT-F), modified Ankylosing Spondylitis Quality of Life (ΔASQoL), Adverse Events (AEs), Serious Adverse Events (SAEs), Upper Respiratory Tract Infection (URTI), nasopharyngitis, diarrhea, as well as headache. Exclusion criteria were: 1) Duplicate publications or results; 2) Reviews or meta-analyses; 3) Conference abstracts or case reports; 4) Animal studies or other non-human research; and 5) Studies lacking extractable outcomes.

### Literature and data extraction

Two researchers independently determined eligible studies as per the predefined eligibility criteria, with a third researcher resolving any disagreements as needed. EndNote 21 software was used for literature management. After partially automated removal of duplicate studies, the rest were filtered through title and abstract checking. Full texts were further reviewed to determine eligible studies for our final analysis. For each encompassed study, key information and primary outcomes were extracted. Basic information encompassed: author names, publication year, design, interventions, sample size, age, as well as sex; primary clinical outcomes encompassed efficacy and safety measures, and risk of bias assessment.

### Quality assessment

The risk of bias was evaluated via the Cochrane Risk of Bias tool (RoB2.0), specifically designed for assessing bias in RCTs.^20^ Bias judgments were made for the randomization process, deviations from intended interventions, missing outcome data, outcome measurement, reported result selection, as well as overall bias, categorized as low risk, some concerns, or high risk. The Newcastle-Ottawa Scale (NOS) was employed to rate cohort study quality^21^ and involved eight items grouped into three dimensions: study population selection, comparability across groups, as well as outcome assessment. Scores range from 0 to 9, with 0‒4, 5–6, and 7‒9 denoting low, moderate, and high quality. Although some of the parent studies were RCTs, these specific analyses involved post hoc, non-randomized subgroup comparisons and were therefore evaluated as cohort studies using the NOS.

### Statistical analysis

Statistical analyses were enabled by Stata 18.0. For dichotomous outcomes, Relative Risk (RR) was employed as the effect measure, whereas for continuous ones, Standardized Mean Difference (SMD) was employed, with all effect sizes shown in 95% Confidence Intervals (95% CI). Heterogeneity was detected via the I^2^ statistic. If I² ≤ 50% and p ≥ 0.1, a Fixed-Effects Model (FEM) was adopted; if I^2^ > 50% and p < 0.1, suggesting substantial heterogeneity, a Random-Effects Model (REM) was utilized. The origins of heterogeneity were found through subgroup analyses (by endpoint and dosage) using the same effect model as in the primary analysis. Sensitivity analyses were performed through a Leave-One-Out (LOO) approach to rate result robustness. Publication bias was rated utilizing funnel plots and Egger’s test, with asymmetry signifying possible bias. If p < 0.05, the trim-and-fill approach was applied to adjust for bias.

## Results

### Study selection and study characteristics

**Fig. 1:**
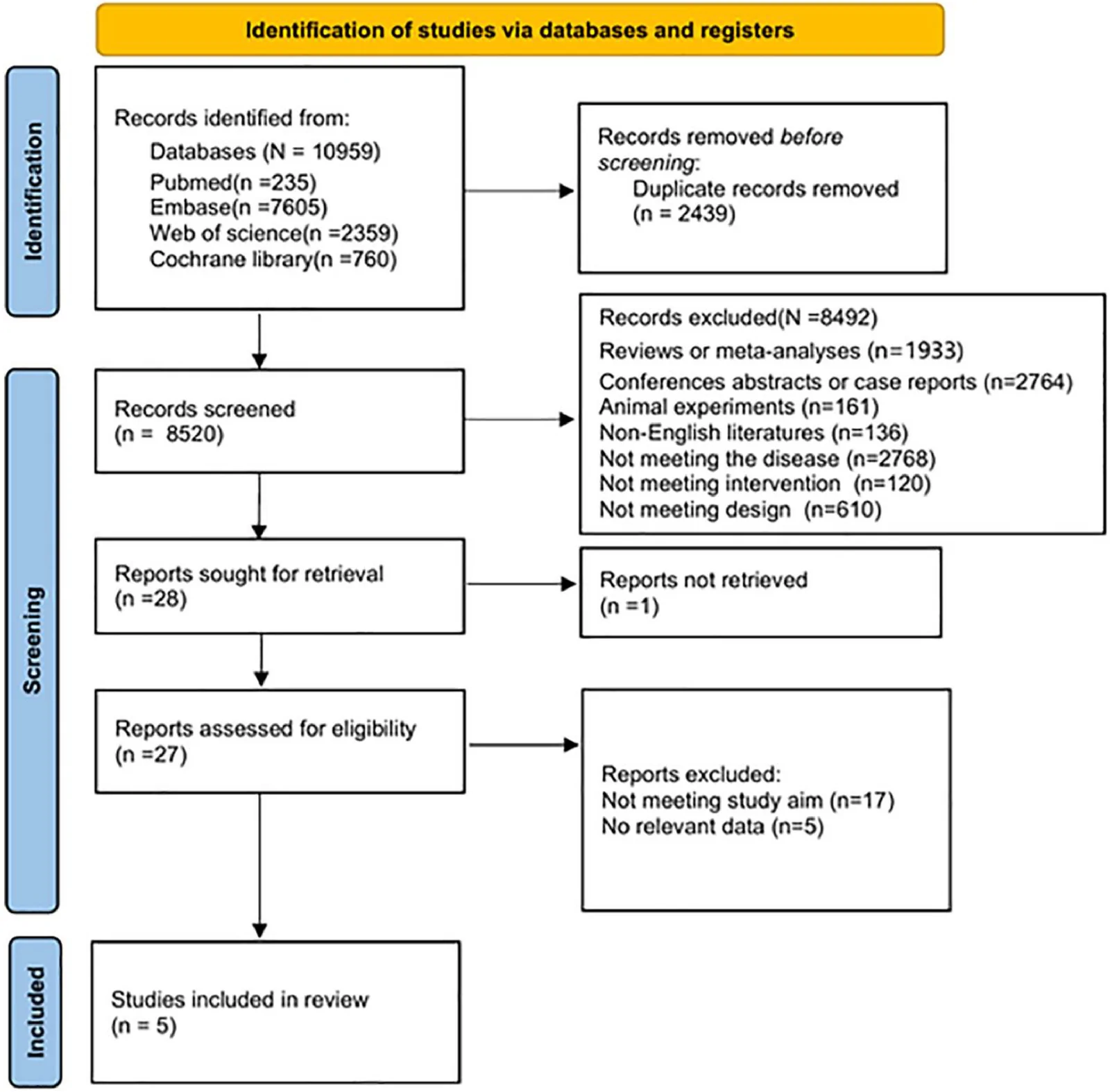
Flow chart of the process used for study identification and selection.

10,959 articles were identified, including 235 from PubMed, 7605 from Embase, 2359 from Web of Science, and 760 from Cochrane Library. Following the removal of 2439 duplicates, 8492 articles were eliminated through title and abstract screening, mostly due to irrelevance or noncompliance with eligibility criteria. Full texts of 27 articles were reviewed, resulting in the exclusion of 22 studies after further evaluation. Ultimately, five studies were encompassed: three RCTs^6^^,^^18^^,^^22^ and two cohort studies.^23^^,^^24^ The process is illustrated in Fig. 1.

Basic characteristics are provided in Appendix 3. All studies encompassed a placebo group and a tofacitinib treatment group, with doses of 2 mg, 5 mg, and 10 mg twice daily, with 5 mg being the primary dose studied. Their mean age of participants was 35.7‒45.8, and they were predominantly young to middle-aged adults, with a higher proportion of males (approximately 60%‒80%), which is consistent with the epidemiology of AS. The primary endpoints were assessed at week 12 or 16, and outcomes broadly covered both efficacy and safety.

### Risk of bias assessment

The risk of bias of the included RCTs is summarized in Appendix 4. For the domain “Selection of the reported result”, all were rated as some concerns, while all were rated as low risk in the other four domains. Therefore, the overall bias of the three studies was judged as some concerns. For the two cohort studies, both received 4-points for study population selection, 1-point for comparability across groups, and 2-points for outcome assessment, yielding a total score of 7, indicating high quality (Appendix 4).

### Meta-analysis results

#### Primary efficacy

**Fig. 2:**
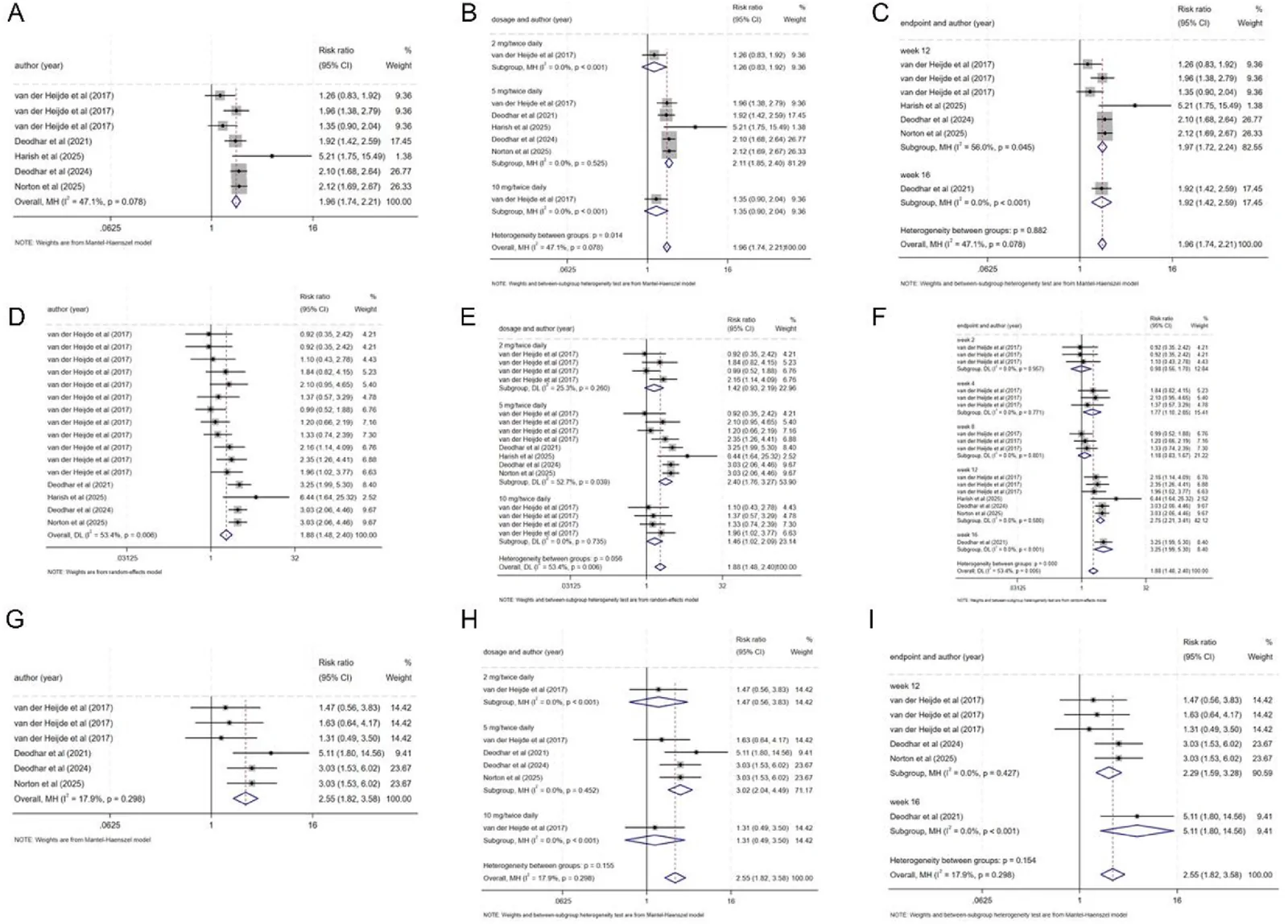
Forest plots of primary efficacy.

The ASAS20 response refers to a 20% improvement as per the ASAS criteria. All five studies reported ASAS20 outcomes, without significant heterogeneity noted (I² = 47.1%, p = 0.078), so a FEM was employed. In contrast to placebo, tofacitinib significantly increased the ASAS20 response rate (RR = 1.96; 95% CI: 1.74 to 2.21). Subgroup analysis indicated that the 5 mg dose group exhibited the most pronounced effect (RR = 2.11, 95% CI: 1.85 to 2.40), with minimal differences observed between the 12-week (RR = 1.97, 95% CI: 1.72 to 2.24) and 16-week assessments (RR = 1.92, 95% CI: 1.42 to 2.59) (Fig. 2A‒C).

Improvement in ASAS40 scores is also a key efficacy endpoint, reflecting a 40% improvement according to the ASAS criteria. All encompassed studies reported ASAS40 outcomes, with moderate heterogeneity detected (I² = 53.4%, p = 0.006); hence, a REM was adopted. Tofacitinib significantly increased the ASAS40 response rate in comparison to placebo (RR = 1.88; 95% CI: 1.48 to 2.40). Dose and assessment time point possibly contribute to heterogeneity. Among different dosing regimens, the 5 mg group showed the greatest efficacy (RR = 2.40, 95% CI: 1.76 to 3.27). Additionally, efficacy tended to improve with prolonged treatment duration, reaching the highest effect at week 16 (RR = 3.25, 95% CI: 1.99 to 5.30) (Fig. 2D‒F). However, this finding was based on a single study and should be interpreted with caution given the wide CI.

Four studies reported ASAS-PR, analyzed using a FEM (I^2^ = 17.9%, p = 0.298). Tofacitinib notably elevated the ASAS-PR response rate in contrast to placebo (RR = 2.55; 95% CI: 1.82 to 3.58). Subgroup analyses indicated the 5 mg dose group had the most marked improvement (RR = 3.02, 95% CI: 2.04 to 4.49), whereas differences were statistically insignificant in the remaining dose groups (p > 0.05). Time-point subgroup analyses indicated superior efficacy at week 16 (RR = 5.11, 95% CI: 1.80 to 14.56) (Fig. 2G‒I).

#### BASDAI50

**Fig. 3:**
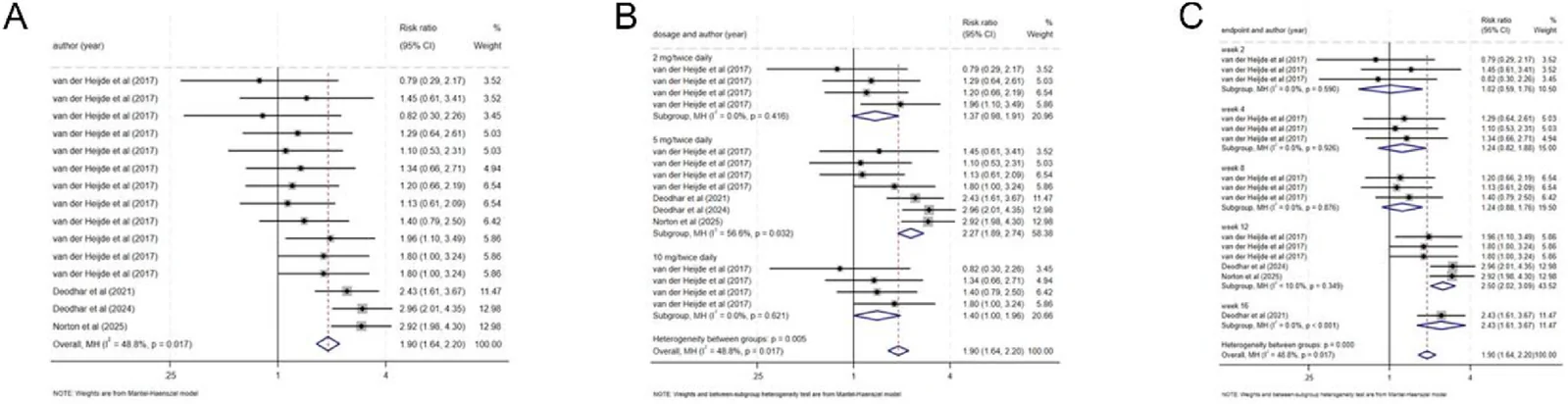
Forest plots of BASDAI50.

BASDAI50 represents a 50% improvement in DA according to specific criteria.^25^ Four studies reported BASDAI50, analyzed using a FEM (I² = 48.8%, p = 0.017). In comparison to placebo, tofacitinib led to a marked rise in BASDAI50 response rate (RR = 1.90; 95% CI: 1.64 to 2.20). Subgroup analyses by dose revealed higher heterogeneity in the 5 mg cohort (I² = 56.6%, p = 0.032), with significant intergroup differences (p = 0.005). Time-point subgroup analyses showed no statistical differences at weeks 2, 4, and 8 (p > 0.05), whereas significant effects were observed at weeks 12 (RR = 2.50, 95% CI: 2.02 to 3.09) and 16 (RR = 2.43, 95% CI: 1.61 to 3.67), with minimal differences between them (Fig. 3A‒C).

#### ASDAS (CRP)

**Fig. 4:**
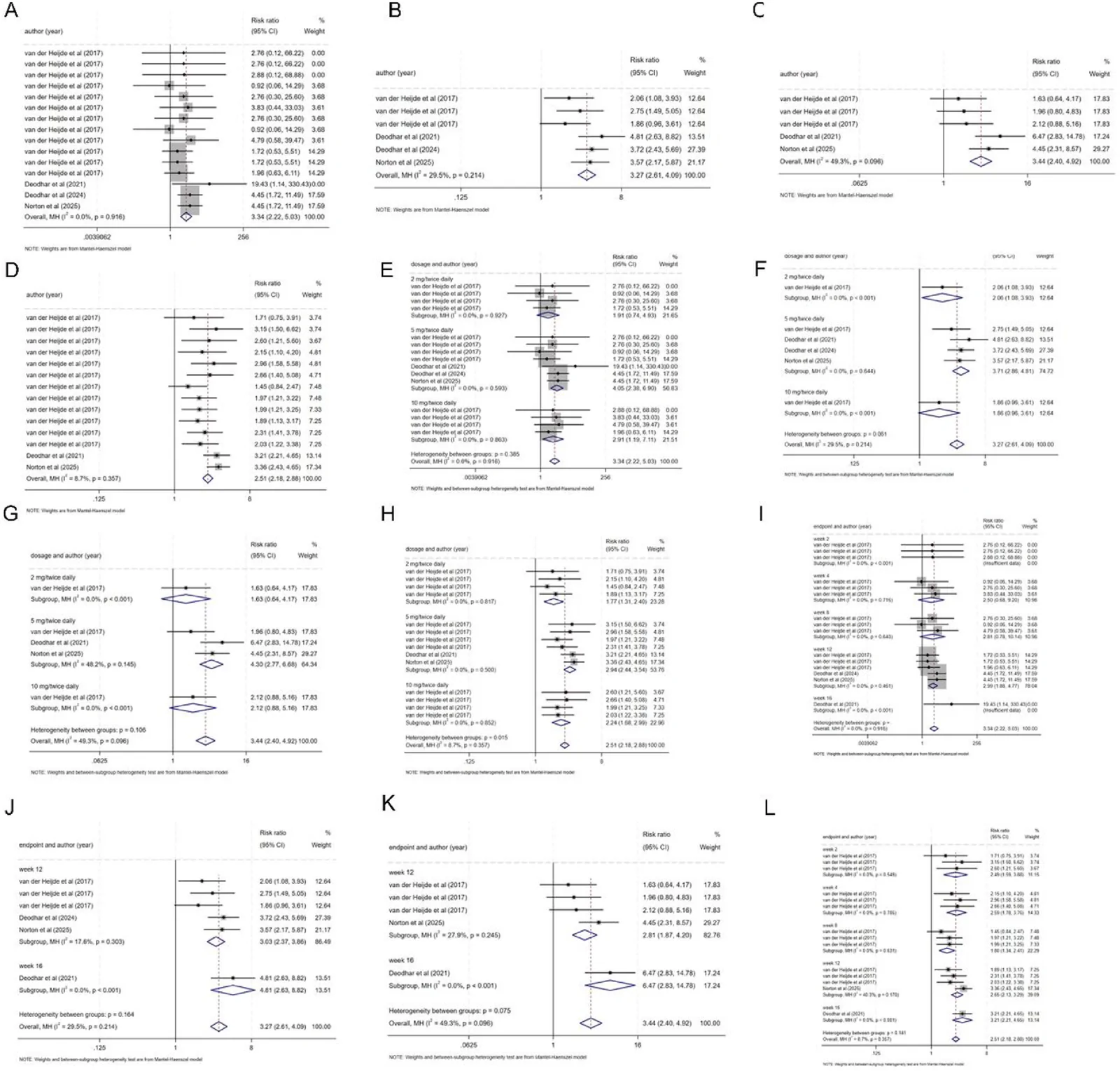
Forest plots of ASDAS.

Four studies reported ASDAS ID (I^2^ = 0%, p = 0.916), three studies reported ASDAS LDA (I^2^ = 29.5%, p = 0.214), ASDAS MR (I^2^ = 49.3%, p = 0.096), and ASDAS CII (I^2^ = 8.7%, p = 0.357), all analyzed using FEMs. The pooled effect sizes were 3.34 (95% CI: 2.22 to 5.03), 3.27 (95% CI: 2.61 to 4.09), 3.44 (95% CI: 2.40 to 4.92), and 2.51 (95% CI: 2.18 to 2.88), respectively, all statistically significant in comparison to the control group (Fig. 4A‒D). Dose subgroup analyses across the four endpoints consistently indicated the 5 mg group demonstrated the most pronounced effects (Fig. 4E‒H), with the greatest efficacy observed at week 16 (Fig. 4I‒L).

#### ΔBASFI and ΔBASMI

**Fig. 5:**
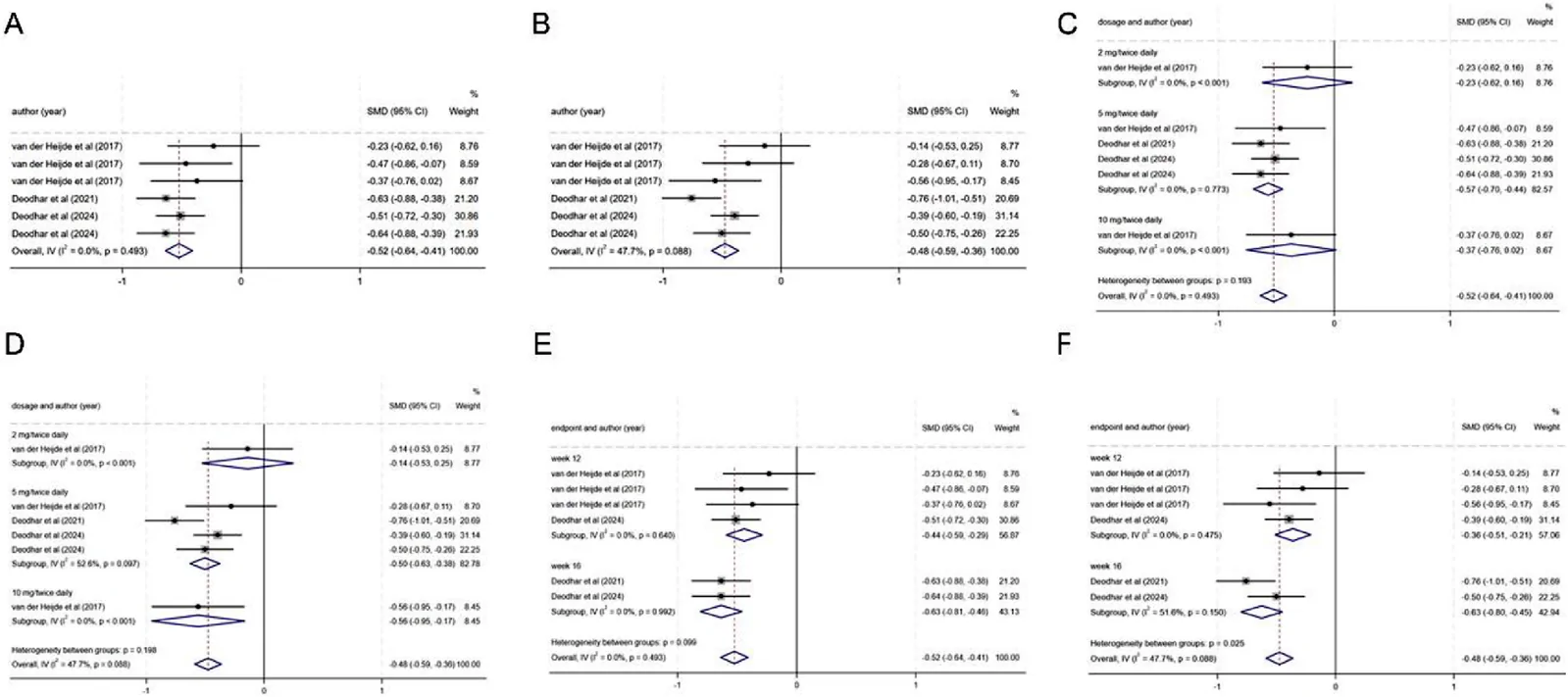
Forest plots of ΔBASFI and ΔBASMI.

Three studies reported ΔBASFI and ΔBASMI, analyzed using FEMs, with I² values of 0% (p = 0.493) and 47.7% (p = 0.088). In contrast to controls, the intervention cohort demonstrated significant improvement, with overall effect sizes of ΔBASFI (SMD = −0.52; 95% CI: −0.64 to −0.41) and ΔBASMI (SMD = −0.48; 95% CI: −0.59 to −0.36) (Fig. 5A‒B). Subgroup analyses showed that for ΔBASFI, the 5 mg dose group had the most prominent effect (SMD = −0.57; 95% CI: −0.70 to −0.44). For ΔBASMI, the 10 mg dose cohort displayed the best improvement effect (SMD = −0.56; 95% CI: −0.95 to −0.17) (Fig. 5C‒D). Furthermore, both endpoints at week 16 demonstrated greater efficacy than at week 12 (Fig. 5E‒F).

#### Quality of life (QoL) and patient-reported outcomes

**Fig. 6:**
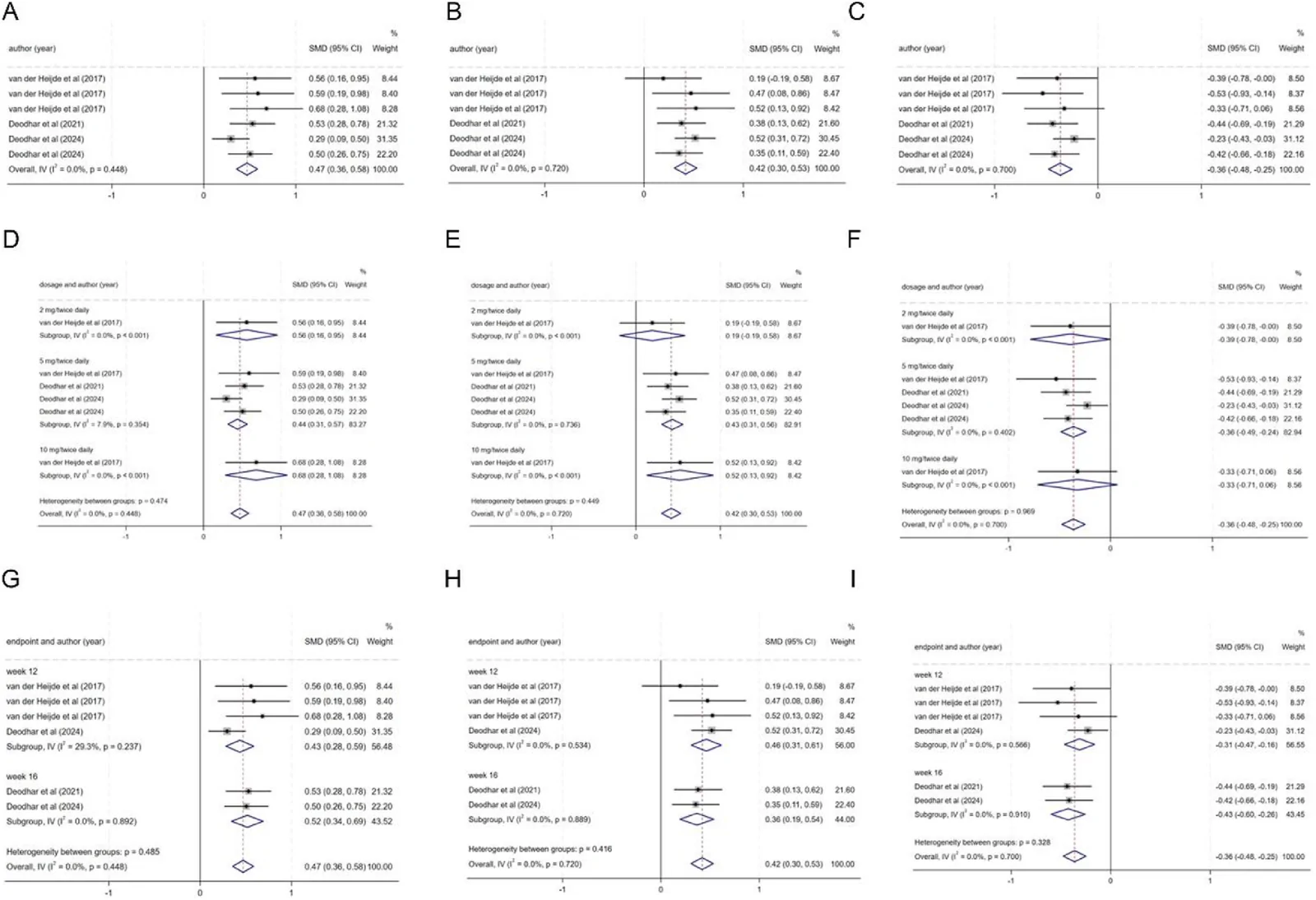
Forest plots of ∆SF-36v2 PCS, ∆FACIT-F and ∆ASQoL.

Three studies reported ∆SF-36v2 PCS, ∆FACIT-F, and ∆ASQoL, with insignificant heterogeneity (I² = 0%; p = 0.448, 0.720, 0.700, respectively), analyzed using FEMs. The intervention group demonstrated significant improvement for all three endpoints, with pooled effect sizes of ∆SF-36v2 PCS (SMD = 0.47, 95% CI: 0.36 to 0.58), ∆FACIT-F (SMD = 0.42, 95% CI: 0.30 to 0.53), and ∆ASQoL (SMD = −0.36, 95% CI: −0.48 to −0.25) (Fig. 6A‒C). Dose subgroup analyses revealed that the 10 mg group showed the greatest improvement in ∆SF-36v2 PCS and ∆FACIT-F, whereas the 5 mg group demonstrated superior improvement in ∆ASQoL compared with the 10 mg group (Fig. 6D‒F). Time-point subgroup analyses indicated that week 12 yielded the greatest effect for ∆FACIT-F, whereas week 16 showed the greatest effect for ∆SF-36v2 PCS and ∆ASQoL (Fig. 6G‒I).

#### AEs

Four studies reported AEs and SAEs, analyzed via FEMs (I^2^ = 0%; p = 0.974, 0.653, respectively). Meta-analysis showed no statistically significant differences across groups. Three studies reported URTI, analyzed using a FEM (I^2^ = 0%, p = 0.493), with a pooled effect size of 2.15 (95% CI: 1.27 to 3.63), revealing a notable increase in incidence in the tofacitinib group. Subgroup analyses showed the 5 mg dose at week 16 had the most pronounced response. Three studies reported nasopharyngitis, diarrhea, and headache, with statistically insignificant differences between groups in the meta-analysis (Appendix 5).

### Sensitivity analysis

A LOO sensitivity analysis was carried out. No single study significantly affected the pooled effect estimates, suggesting robust stability of the findings (Appendix 6).

### Publication bias

Publication bias was detected through funnel plots and Egger’s test (Appendix 7). Significant publication bias was not noted for ASAS20 (p = 0.385), ASAS40 (p = 0.580), BASDAI50 (p = 0.113), ASDAS LDA (p = 0.143), ASDAS MR (p = 0.204), ΔBASFI (p = 0.136), ΔBASMI (p = 0.539), ∆SF-36v2 PCS (p = 0.061), ∆FACIT-F (p = 0.618), and ∆ASQoL (p = 0.306) (p > 0.05). However, outcomes ASAS-PR (p = 0.004), ASDAS ID (p = 0.000), as well as ASDAS CII (p = 0.001) suggested potential publication bias. Trim-and-fill analyses indicated that although some publication bias existed for ASAS-PR, ASDAS ID, and ASDAS CII, the overall reliability of the results was not compromised.

## Discussion

This study provides a comprehensive and up-to-date systematic review and meta-analysis on the efficacy and safety of tofacitinib in managing the AS population. Five studies were encompassed (three RCTs and two cohort studies), and the synthesized evidence indicates that tofacitinib shows notable efficacy in improving multiple clinical, functional, and patient-reported outcomes in the AS population, with an overall acceptable safety profile.

Firstly, regarding primary efficacy endpoints, compared with placebo, tofacitinib significantly increased response rates of ASAS20, ASAS40, and ASAS-PR, suggesting that tofacitinib not only facilitates the attainment of initial clinical improvement but also enables a substantial proportion of patients to achieve deep disease remission. Notably, there was considerable heterogeneity in ASAS40 outcomes, and subgroup analyses suggested that dosing and treatment duration possibly be key sources of heterogeneity. The 5 mg dose group demonstrated the most favorable efficacy trend across primary efficacy endpoints, with therapeutic effects enhanced over time and peaking at 16-weeks. Given that the 10 mg dose did not consistently outperform 5 mg in these primary efficacy outcomes and is not currently approved for AS treatment, a dosage of 5 mg administered twice daily is likely the optimal regimen balancing efficacy and safety. This strongly implies that optimal deep remission possibly requires both sufficient dosing (5 mg BID) and adequate treatment duration (at least 16-weeks).

Secondly, regarding DA indices, tofacitinib consistently and significantly improved BASDAI50 and ASDAS ID, LDA, MR, and CII, which aligns with findings from a similar meta-analysis.^26^ Tofacitinib possibly exerts its therapeutic effect by directly or indirectly inhibiting multiple pro-inflammatory cytokine signaling pathways like IL-6, IL-23, and IL-17, and inhibiting the activation and function of immune cells like NK and T-cells, thereby modulating immune-inflammatory responses and alleviating clinical symptoms.^27^

Moreover, a key highlight of our study is the comprehensive assessment of tofacitinib on patients’ Physical Function (PF) and QoL. Significant improvements in ΔBASFI and ΔBASMI indicate that treatment translates directly into enhanced daily functioning and spinal mobility, which are crucial for AS patients to maintain work capacity and independent living. Additionally, in terms of QoL measures, tofacitinib led to clinically meaningful improvements across general health surveys (e.g., SF-36 PCS), fatigue-specific scales (e.g., FACIT-F), as well as disease-specific QoL instruments (e.g., ASQoL). Notably, while the 5 mg dose showed the most favorable performance in primary efficacy outcomes, the 10 mg dose also demonstrated effectiveness in these PF (e.g., ΔBASMI) and QoL (e.g., ∆SF-36v2 PCS and ∆FACIT-F) outcomes. This clearly demonstrates that its therapeutic effects extend beyond laboratory and physician-assessed measures, reflecting real improvements in patient-perceived health status, fatigue, and overall life satisfaction, marking a shift from a “biomedical” to a “bio-psycho-social” model of medical care. However, given that the 10 mg dose did not consistently outperform 5 mg across all measures and is not approved for the treatment of AS, the 5 mg twice-daily regimen remains the preferred clinical choice.

Regarding safety outcomes, the overall incidence of AEs, SAEs, and common side effects like nasopharyngitis, diarrhea, and headache did not differ significantly from placebo, consistent with previous safety studies by Li^26^ and Lee,^28^ suggesting relative safety in AS patients. However, a signal warranting caution is the significantly increased risk of URTI, which aligns with findings in psoriasis studies^29^ and possibly relates to the broad immunosuppressive effect of tofacitinib.^27^ Subgroup analyses indicated that this risk was more pronounced at 5 mg and 16-weeks, paralleling efficacy trends, suggesting a potential relation of therapeutic effect to infection risk.

Our strengths lie in strict adherence to systematic review and meta-analysis reporting standards; thorough searches of multiple major databases to minimize omissions; evaluation of not only core efficacy endpoints but also function, QoL, and multiple AEs; detailed subgroup analyses (dose, duration) to explore sources of heterogeneity and optimal treatment strategies; and rigorous risk-of-bias assessment, sensitivity analyses, and publication bias evaluation, ensuring robustness of results.

Nevertheless, several limitations exist. First, the encompassed studies were limited (only five), potentially affecting the statistical power of certain subgroup analyses. In particular, although the findings from these subgroup analyses may provide useful insights, they should be interpreted with caution given the limited number of studies included in each subgroup. Second, treatment durations in the encompassed studies were predominantly 12‒16 weeks, lacking long-term efficacy and safety data, which is especially relevant for AS, a chronic disease requiring lifelong management. Notably, this limited duration precluded the assessment of long-term safety signals, particularly the risk of Major Adverse Cardiovascular Events (MACE), malignancies, and thromboembolic events, which are recognized safety concerns associated with JAK inhibitors and were highlighted in the ORAL Surveillance trial for rheumatoid arthritis.^30^ Therefore, although large-scale studies with long-term follow-up are urgently needed to confirm long-term efficacy, characterize drug resistance, and identify rare SAEs, we recommend that clinicians perform baseline risk assessments, including cardiovascular and thromboembolic evaluations, and conduct regular laboratory monitoring throughout treatment to mitigate potential safety concerns in clinical practice. Third, although subgroup analyses were carried out to find the origins of heterogeneity, moderate heterogeneity remained for some outcomes (e.g., ASAS40, BASDAI50), the precise causes of which remain unclear and possibly relate to unmeasured study or patient characteristics. Additionally, according to the RoB2.0 assessment, all included RCTs were judged to have “some concerns” in the domain of “selection of the reported result”, indicating a potential risk of reporting bias that should be taken into account when interpreting the findings.

## Conclusion

Tofacitinib demonstrates significant efficacy in active AS, improving DA, PF, and QoL, with overall manageable safety, the primary risk being an increased yet controllable risk of URTI. For the population with active AS inadequately responsive or intolerant to NSAIDs or bDMARDs, tofacitinib offers an effective oral targeted therapeutic option. Based on the available data, the 5 mg twice-daily dose appears to be the optimal regimen for the management of AS, with treatment effects becoming more pronounced after 12‒16 weeks. Future research should focus on: 1) Performing longer-term, larger-scale RCTs and real-world studies to confirm long-term efficacy and safety; 2) Exploring combination therapy strategies with other agents, like NSAIDs, conventional DMARDs, or other biologics; and 3) Identifying biomarkers predicting efficacy and AEs to enable individualized precision therapy.

## Clinical implications

This meta-analysis provides clinically meaningful evidence supporting tofacitinib as an effective oral targeted therapy for patients with active AS who have an inadequate response to or are intolerant of conventional treatments. The 5-mg twice-daily regimen appears to offer the most favorable balance between efficacy and safety. However, sustained treatment may be necessary to achieve optimal clinical benefit, with therapeutic effects becoming more evident after 12‒16 weeks. Although the overall safety profile is acceptable, clinicians should remain vigilant for URTIs, perform baseline risk assessments, including cardiovascular and thromboembolic evaluations, and conduct regular laboratory monitoring throughout treatment to mitigate potential safety concerns. Further large-scale studies with long-term follow-up are warranted to establish the durability of efficacy, characterize long-term safety, and identify biomarkers that may facilitate personalized treatment strategies.

## Ethics approval

Not applicable.

## Consent to participate

Not applicable.

## Consent to publish

Not applicable.

## Clinic trial number

Not applicable.

## Authors’ contributions

Le Shen: Conceptualization; Methodology; Investigation; Software; Data curation. Fei Huang: Investigation; Data curation; Visualization; Writing-original draft preparation. Kang Han: Validation; Supervision; Funding acquisition. Lei Bo: Writing-reviewing and Editing, and all authors commented on previous versions of the manuscript. All authors read and approved the final manuscript.

## Funding

This research was supported by the National Natural Science Foundation of China (NSFC) (81,702,935), the Natural Science Foundation of Shandong Provincial (ZR2023MH206), and the Project of Medical and Health Science and Technology Development Program of Shandong province (202,204,071,065).

## Data availability statement

All data generated or analyzed during this study are included in this published article and its supplementary information files.

## Declaration of competing interest

The authors declare no conflicts of interest.
