# Supplementary material for: Efficacy and safety of tofacitinib in ankylosing spondylitis patients: a systematic review and meta-analysis
Source: Clinics (Sao Paulo). 2026 Sep 9;81:101147. doi: 10.1016/j.clinsp.2026.101147 (PMC13573708; doi:10.1016/j.clinsp.2026.101147)
Supplement: Supplementary file 1 [file mmc1.docx]

Appendix 2 Search strategy

PubMed

| Search number | Query | Search details | Results |
| --- | --- | --- | --- |
| 1 | "Spondylitis, Ankylosing"[Mesh] | "spondylitis, ankylosing"[MeSH Terms] | 17253 |
| 2 | "ankylating spondylitis"[Title/Abstract] OR "ankylopoietic spondylarthritis"[Title/Abstract] OR "ankylopoietic spondylitis"[Title/Abstract] OR "ankylosing spine"[Title/Abstract] OR "ankylosing spondilitis"[Title/Abstract] OR "ankylosing spondylarthr*"[Title/Abstract] OR "Ankylosing Spondylitis"[Title/Abstract] OR "Ankylosing Spondyloarthriti*"[Title/Abstract] OR "ankylosis spondylitis"[Title/Abstract] OR "ankylotic spondylitis"[Title/Abstract] OR "AS"[Title/Abstract] OR "Bechterew* disease"[Title/Abstract] OR "bekhterev disease"[Title/Abstract] OR "Marie Struempell Disease"[Title/Abstract] OR "morbus bechterew"[Title/Abstract] OR "Rheumatoid Spondylitis"[Title/Abstract] OR "spinal ankylosis"[Title/Abstract] OR "spine ankylosis"[Title/Abstract] OR "spondylarthr* ankylopoietica"[Title/Abstract] OR "spondylarthritis ankylosans"[Title/Abstract] OR "spondylitis ankylopo*"[Title/Abstract] OR "spondyloarthritis ankylopoietica"[Title/Abstract] OR "vertebral ankylosis"[Title/Abstract] | "ankylopoietic spondylarthritis"[Title/Abstract] OR "ankylopoietic spondylitis"[Title/Abstract] OR "ankylosing spine"[Title/Abstract] OR "ankylosing spondilitis"[Title/Abstract] OR "ankylosing spondylarthr*"[Title/Abstract] OR "Ankylosing Spondylitis"[Title/Abstract] OR "ankylosing spondyloarthriti*"[Title/Abstract] OR "ankylosis spondylitis"[Title/Abstract] OR "AS"[Title/Abstract] OR "bechterew* disease"[Title/Abstract] OR "Marie Struempell Disease"[Title/Abstract] OR "morbus bechterew"[Title/Abstract] OR "Rheumatoid Spondylitis"[Title/Abstract] OR "spinal ankylosis"[Title/Abstract] OR "spine ankylosis"[Title/Abstract] OR "spondylarthr* ankylopoietica"[Title/Abstract] OR "spondylitis ankylopo*"[Title/Abstract] OR "spondyloarthritis ankylopoietica"[Title/Abstract] OR "vertebral ankylosis"[Title/Abstract] | 1204355 |
| 3 | #1 OR #2 | "spondylitis, ankylosing"[MeSH Terms] OR "ankylopoietic spondylarthritis"[Title/Abstract] OR "ankylopoietic spondylitis"[Title/Abstract] OR "ankylosing spine"[Title/Abstract] OR "ankylosing spondilitis"[Title/Abstract] OR "ankylosing spondylarthr*"[Title/Abstract] OR "Ankylosing Spondylitis"[Title/Abstract] OR "ankylosing spondyloarthriti*"[Title/Abstract] OR "ankylosis spondylitis"[Title/Abstract] OR "AS"[Title/Abstract] OR "bechterew* disease"[Title/Abstract] OR "Marie Struempell Disease"[Title/Abstract] OR "morbus bechterew"[Title/Abstract] OR "Rheumatoid Spondylitis"[Title/Abstract] OR "spinal ankylosis"[Title/Abstract] OR "spine ankylosis"[Title/Abstract] OR "spondylarthr* ankylopoietica"[Title/Abstract] OR "spondylitis ankylopo*"[Title/Abstract] OR "spondyloarthritis ankylopoietica"[Title/Abstract] OR "vertebral ankylosis"[Title/Abstract] | 1208549 |
| 4 | "tofacitinib" [Supplementary Concept] | "tofacitinib"[Supplementary Concept] | 1951 |
| 5 | "jaquinus"[Title/Abstract] OR "tasocitinib"[Title/Abstract] OR "tofacitinib"[Title/Abstract] OR "Xeljanz"[Title/Abstract] | "tasocitinib"[Title/Abstract] OR "tofacitinib"[Title/Abstract] OR "Xeljanz"[Title/Abstract] | 3685 |
| 6 | #4 OR #5 | "tofacitinib"[Supplementary Concept] OR "tasocitinib"[Title/Abstract] OR "tofacitinib"[Title/Abstract] OR "Xeljanz"[Title/Abstract] | 3890 |
| 7 | #3 AND #6 | ("spondylitis, ankylosing"[MeSH Terms] OR ("ankylopoietic spondylarthritis"[Title/Abstract] OR "ankylopoietic spondylitis"[Title/Abstract] OR "ankylosing spine"[Title/Abstract] OR "ankylosing spondilitis"[Title/Abstract] OR "ankylosing spondylarthr*"[Title/Abstract] OR "Ankylosing Spondylitis"[Title/Abstract] OR "ankylosing spondyloarthriti*"[Title/Abstract] OR "ankylosis spondylitis"[Title/Abstract] OR "AS"[Title/Abstract] OR "bechterew* disease"[Title/Abstract] OR "Marie Struempell Disease"[Title/Abstract] OR "morbus bechterew"[Title/Abstract] OR "Rheumatoid Spondylitis"[Title/Abstract] OR "spinal ankylosis"[Title/Abstract] OR "spine ankylosis"[Title/Abstract] OR "spondylarthr* ankylopoietica"[Title/Abstract] OR "spondylitis ankylopo*"[Title/Abstract] OR "spondyloarthritis ankylopoietica"[Title/Abstract] OR "vertebral ankylosis"[Title/Abstract])) AND ("tofacitinib"[Supplementary Concept] OR ("tasocitinib"[Title/Abstract] OR "tofacitinib"[Title/Abstract] OR "Xeljanz"[Title/Abstract])) | 235 |

Embase

| No. | Query | Results |
| --- | --- | --- |
| #1 | 'ankylosing spondylitis'/exp | 37096 |
| #2 | 'ankylating spondylitis':ti,ab,kw OR 'ankylopoietic spondylarthritis':ti,ab,kw OR 'ankylopoietic spondylitis':ti,ab,kw OR 'ankylosing spine':ti,ab,kw OR 'ankylosing spondilitis':ti,ab,kw OR 'ankylosing spondylarthr*':ti,ab,kw OR 'ankylosing spondylitis':ti,ab,kw OR 'ankylosing spondyloarthriti*':ti,ab,kw OR 'ankylosis spondylitis':ti,ab,kw OR 'ankylotic spondylitis':ti,ab,kw OR 'as':ti,ab,kw OR 'bechterew* disease':ti,ab,kw OR 'bekhterev disease':ti,ab,kw OR 'marie struempell disease':ti,ab,kw OR 'morbus bechterew':ti,ab,kw OR 'rheumatoid spondylitis':ti,ab,kw OR 'spinal ankylosis':ti,ab,kw OR 'spine ankylosis':ti,ab,kw OR 'spondylarthr* ankylopoietica':ti,ab,kw OR 'spondylarthritis ankylosans':ti,ab,kw OR 'spondylitis ankylopo*':ti,ab,kw OR 'spondyloarthritis ankylopoietica':ti,ab,kw OR 'vertebral ankylosis':ti,ab,kw | 18946979 |
| #3 | #1 OR #2 | 18954394 |
| #4 | 'tofacitinib'/exp | 13201 |
| #5 | 'jaquinus':ti,ab,kw OR 'tasocitinib':ti,ab,kw OR 'tofacitinib':ti,ab,kw OR 'xeljanz':ti,ab,kw | 7588 |
| #6 | #4 OR #5 | 13583 |
| #7 | #3 AND #6 | 7605 |

Web of Science

| # | Search Query | Results |
| --- | --- | --- |
| 14 | TS=((ankylating spondylitis) OR (ankylopoietic spondylarthritis) OR (ankylopoietic spondylitis) OR (ankylosing spine) OR (ankylosing spondilitis) OR (ankylosing spondylarthr*) OR (Ankylosing Spondylitis) OR (Ankylosing Spondyloarthriti*) OR (ankylosis spondylitis) OR (ankylotic spondylitis) OR (AS) OR (Bechterew* disease) OR (bekhterev disease) OR (Marie Struempell Disease) OR (morbus bechterew) OR (Rheumatoid Spondylitis) OR (spinal ankylosis) OR (spine ankylosis) OR (spondylarthr* ankylopoietica) OR (spondylarthritis ankylosans) OR (spondylitis ankylopo*) OR (spondyloarthritis ankylopoietica) OR (vertebral ankylosis)) | 24483571 |
| 15 | TS=((jaquinus) OR (tasocitinib) OR (tofacitinib) OR (Xeljanz)) | 6045 |
| 16 | #15 AND #14 | 2359 |

Cochrane Library

| ID | Search | Hits |
| --- | --- | --- |
| #1 | MeSH descriptor: [Spondylitis, Ankylosing] explode all trees | 942 |
| #2 | (‘ankylating spondylitis’ OR ‘ankylopoietic spondylarthritis’ OR ‘ankylopoietic spondylitis’ OR ‘ankylosing spine’ OR ‘ankylosing spondilitis’ OR ‘ankylosing spondylarthr*’ OR ‘Ankylosing Spondylitis’ OR ‘Ankylosing Spondyloarthriti*’ OR ‘ankylosis spondylitis’ OR ‘ankylotic spondylitis’ OR ‘AS’ OR ‘Bechterew* disease’ OR ‘bekhterev disease’ OR ‘Marie Struempell Disease’ OR ‘morbus bechterew’ OR ‘Rheumatoid Spondylitis’ OR ‘spinal ankylosis’ OR ‘spine ankylosis’ OR ‘spondylarthr* ankylopoietica’ OR ‘spondylarthritis ankylosans’ OR ‘spondylitis ankylopo*’ OR ‘spondyloarthritis ankylopoietica’ OR ‘vertebral ankylosis’):ti,ab,kw | 1002063 |
| #3 | #1 OR #2 | 1002063 |
| #4 | (‘jaquinus’ OR ‘tasocitinib’ OR ‘tofacitinib’ OR ‘Xeljanz’):ti,ab,kw | 1255 |
| #5 | #3 AND #4 | 771 |
