# Supplementary material for: Efficacy and safety of tofacitinib in ankylosing spondylitis patients: a systematic review and meta-analysis
Source: Clinics (Sao Paulo). 2026 Sep 9;81:101147. doi: 10.1016/j.clinsp.2026.101147 (PMC13573708; doi:10.1016/j.clinsp.2026.101147)
Supplement: Supplementary file 2 [file mmc2.docx]

**Appendix 3 Baseline characteristics of the included studies**

| Author year | Study type | Group | Number | Age, years (Mean) | Male | Endpoint | Outcome | Risk of bias |
| --- | --- | --- | --- | --- | --- | --- | --- | --- |
| van der Heijde 2017 | RCT | Placebo | 51 | 41.9 | 32 | Week 12 | ASAS20, ASAS40, ASASPR, BASDAI50, ASDAS ID, ASDAS LDA, ASDAS MR , ASDAS CII, ΔBASFI, ∆BASMI, ∆SF-36v2 PCS, ∆FACIT-F, ∆ASQoL, AE, SAE, upper respiratory tract infection, Nasopharyngitis, Diarrhoea, Headache | Some concerns |
|  |  | Tofacitinib 2mg, bid | 52 | 41.8 | 34 |  |  |  |
|  |  | Tofacitinib 5mg, bid | 52 | 41.2 | 39 |  |  |  |
|  |  | Tofacitinib 10mg, bid | 52 | 41.6 | 38 |  |  |  |
| Deodhar2021 | RCT | Placebo | 136 | 40 | 108 | Week 16 | ASAS20, ASAS40, ASASPR, BASDAI50, ASDAS ID, ASDAS LDA, ASDAS MR, ASDAS CII, ΔBASFI, ∆BASMI, ∆SF-36v2 PCS, ∆FACIT-F, ∆ASQoL, AE, SAE, upper respiratory tract infection, Nasopharyngitis, Diarrhoea, Headache | Some concerns |
|  |  | Tofacitinib 5mg, bid | 133 | 42.2 | 116 |  |  |  |
| Harish 2025 | RCT | Placebo | 23 | 36 | 17 | Week 12 | ASAS20, ASAS40, upper respiratory tract infection, Diarrhoea | Some concerns |
|  |  | Tofacitinib 5mg, bid | 25 | 36.5 | 21 |  |  |  |
| Deodhar2024 | Cohort  study | Placebo（CRP<5） | 55 | 42.8 | 37 | Week 12 | ASAS20, ASAS40, ASASPR, BASDAI50, ASDAS ID, ASDAS LDA, ΔBASFI, ∆BASMI, ∆SF-36v2 PCS, ∆FACIT-F, ∆ASQoL, AE, SAE | 7 points |
|  |  | Tofacitinib 5mg, bid (CRP<5） | 58 | 44.7 | 42 |  |  |  |
|  |  | Placebo  (CRP≥5) | 132 | 39.6 | 103 |  |  |  |
|  |  | Tofacitinib 5mg, bid (CRP≥5) | 127 | 40.6 | 113 |  |  |  |
| Norton 2025 | Cohort  study | Placebo（BMI<25） | 79 | 35.7 | 57 | Week 12 | ASAS20, ASAS40, ASASPR, BASDAI50, ASDAS ID, ASDAS LDA, ASDAS MR, ASDAS CII, AE, SAE, upper respiratory tract infection, Nasopharyngitis, Headache | 7 points |
|  |  | Tofacitinib 5mg, bid (BMI<25） | 74 | 36.6 | 60 |  |  |  |
|  |  | Placebo  (25≤BMI<30) | 62 | 42.9 | 51 |  |  |  |
|  |  | Tofacitinib 5mg, bid (25≤BMI<30) | 69 | 44.9 | 60 |  |  |  |
|  |  | Placebo  （BMI≥30） | 45 | 45.7 | 31 |  |  |  |
|  |  | Tofacitinib 5mg, bid（BMI≥30） | 41 | 45.8 | 34 |  |  |  |
