# Supplementary material for: Efficacy and safety of tofacitinib in ankylosing spondylitis patients: a systematic review and meta-analysis
Source: Clinics (Sao Paulo). 2026 Sep 9;81:101147. doi: 10.1016/j.clinsp.2026.101147 (PMC13573708; doi:10.1016/j.clinsp.2026.101147)
Supplement: Supplementary file 3 [file mmc3.docx]

**Appendix 4 Quality assessment of the included studies**

Risk of bias graph for randomized controlled trials using RoB 2.0

| Study | Representativeness of the exposed cohort | Selection of the non exposed cohort | Ascertainment of exposure | Demonstration that outcome of interest was not present at start of study | Comparability of cohorts on the basis of the design or analysis | Assessment of outcome | Was follow-up long enough for outcomes to occur | Adequacy of follow up of cohorts | Total score | Quality assessment |
| --- | --- | --- | --- | --- | --- | --- | --- | --- | --- | --- |
| Deodhar 2024 | 1 | 1 | 1 | 1 | 1 | o.5 | 1 | 0.5 | 7 | Good |
| Norton 2025 | 1 | 1 | 1 | 1 | 1 | o.5 | 1 | o.5 | 7 | Good |

The quality assessment of cohort studies using NOS
