# Supplementary material for: Efficacy and safety of tofacitinib in ankylosing spondylitis patients: a systematic review and meta-analysis
Source: Clinics (Sao Paulo). 2026 Sep 9;81:101147. doi: 10.1016/j.clinsp.2026.101147 (PMC13573708; doi:10.1016/j.clinsp.2026.101147)
Supplement: Supplementary file 5 [file mmc5.docx]

**Appendix 6 Sensitivity analysis**

ASAS20

ASAS40

ASASPR

BASDAI50

ASDAS ID

ASDAS LDA

ASDAS MR

ASDAS CII

ΔBASFI

ΔBASMI

ΔSF-36v2 PCS

ΔFACIT-F

ΔASQoL

URTI
