# Supplementary material for: Efficacy and safety of tofacitinib in ankylosing spondylitis patients: a systematic review and meta-analysis
Source: Clinics (Sao Paulo). 2026 Sep 9;81:101147. doi: 10.1016/j.clinsp.2026.101147 (PMC13573708; doi:10.1016/j.clinsp.2026.101147)
Supplement: Supplementary file 6 [file mmc6.docx]

**Appendix 7 Publication bias**

ASAS20

ASAS40

BSDAI50

ASDAS LDA

ASDAS MR

ΔBASFI

ΔBASMI

∆SF-36v2 PCS

∆FACTT-F

∆ASQoL

ASASPR

ASDAS ID

ASDAS CII
