# Supplementary material for: Efficacy and safety of tofacitinib in ankylosing spondylitis patients: a systematic review and meta-analysis
Source: Clinics (Sao Paulo). 2026 Sep 9;81:101147. doi: 10.1016/j.clinsp.2026.101147 (PMC13573708; doi:10.1016/j.clinsp.2026.101147)
Supplement: Supplementary file 8 [file mmc4.docx]

**Appendix 5 Forest plots of safety**

AE

SAE

URTI

Nasopharyngitis

Diarrhea

Headache
